# Judicial demand of medications through the Federal Justice of the State of Paraná

**DOI:** 10.1590/S1679-45082017GS3792

**Published:** 2017

**Authors:** Renato Mitsunori Nisihara, Ana Carolina Possebom, Luiza de Martino Cruvinel Borges, Ana Claudia Athanasio Shwetz, Fernanda Francis Benevides Bettes

**Affiliations:** 1 Universidade Positivo, Curitiba, PR, Brazil.

**Keywords:** Drug costs/legislation & jurisprudence, Pharmaceutical services/legislation & jurisprudence, Resource allocation/legislation & jurisprudence, Public health/legislation & jurisprudence

## Abstract

**Objective:**

To describe the profile of lawsuits related to drug requests filled at the Federal Justice of the State of Paraná.

**Methods:**

A cross-sectional study, and the data were obtained through consulting the lawsuits at the online system of the Federal Justice of Paraná.

**Results:**

Out of 347 lawsuits included in the study, 55% of plaintiffs were women, with a median age of 56 years. Oncology was the field with more requests (23.6%), and the highest mean costs. A wide variety of diseases and broad variety of requested drugs were found in the lawsuits. Approximately two-thirds of them were requested by the brand name, and the most often requested drugs were palivizumab and tiotropium bromide. Only 14.5% of the requested medicines were registered in the National Medication Register. The Public Defender’s Office filled actions in 89.6% of cases and all lawsuits had an interim relief. The mean time for approval was 35 days and 70% of requests were granted.

**Conclusion:**

Oncology was the field with the highest demand for medicines at the Federal Justice of Paraná in 2014. A great variety of medications was requested. The Public Defender´s Office represented most lawsuits. All demands had an interim relief, and the majority of requests were granted, within an average of 35 days.

## RESUMO

**Objetivo:**

Descrever o perfil das ações que solicitam medicamentos ajuizadas na Justiça Federal do Paraná.

**Métodos:**

Estudo transversal descritivo, cujos dados foram obtidos por meio de consulta aos processos no sistema *on-line* da Justiça Federal do Paraná.

**Resultados:**

Dentre os 347 processos incluídos no estudo, 55% dos autores eram mulheres, com mediana da idade de 56 anos, sendo a área mais procurada a oncologia (23,6%). A área oncológica também foi a que apresentou maiores custos médios. Foi ampla a variedade de doenças geradoras das ações e também foi consequentemente grande a variedade de medicamentos solicitados. Cerca de dois terços dos fármacos foram solicitados pelo nome comercial, e os mais requeridos foram o palivizumabe e brometo de tiotrópio. Apenas 14,5% dos medicamentos solicitados estavam cadastrados no Registro Nacional de Medicamentos. A Defensoria Pública impetrou as ações em 89,6% dos casos. Todos os processos pediam antecipação de tutela do medicamento. O tempo médio para deferimento foi de 35 dias, sendo que 70% dos pedidos foram deferidos.

**Conclusão:**

A área com maior número de casos de demanda de medicamentos na Justiça Federal do Paraná no ano de 2014 foi de Oncologia. Observou-se grande variedade de medicamentos solicitados. A maioria das ações foi impetrada pela Defensoria Pública. Todas as demandas exigiram antecipação de tutela, sendo que a maioria dos pedidos foram deferidos, num prazo médio de 35 dias.

## INTRODUCTION

The purpose of the Unified Health System (*Sistema Único de Saúde* – SUS) is to grant access of the Brazilian population to the health system, in a comprehensive, universal and equal manner, including outpatient services and organ transplantation.^1^ To date, 80% of Brazilians are estimated to benefit from the services offered by SUS as regards health care.^2^ The three cornerstones on which SUS is based encourage the strategy of industries to create a market for their new products.

The new reality of regulation for the acquisition of medications has been compromised in the past few years, due to a recent and dramatic increase in lawsuits related to drug requests to health managers, thus configuring what is currently known as “judicialization of the pharmaceutical demand”.^3,4^ Evidence-based studies on the judicialization of health policies in Brazil have shown that the main judicialized items in courts are medications.^5^ Demands for the access to expensive medications, or even to medications not approved by the Brazilian Health Regulatory Agency (*Agencia Nacional de Vigilância Sanitária* – ANVISA) are in a subgroup of judicialization of medications, compromising the principles that guide the national policies on medications, namely safety, efficacy and quality.^6,7^

A large part of this judicial demand comes from requests of medications that are not first distributed by some of the medication assistance programs by the SUS.^8^ As the lawsuits filed by patients force a free medication supply, under a claim of constitutional right to health, the high costs of such medications have an impact on pharmaceutical care provided by SUS.^8^

This impact results from two components of the pharmaceutical industry – the variable and the fixed financial components. The latter consists of the *per capita* value directly coming from the Federal Government; the former has a *per capita* value from a decentralized source, that is, from resources that could come from the Ministry of Health or from municipal departments of health. Therefore, the variable component depends not only on services included in the public health programs but also on those not programed, thus becoming a burden to SUS.^9,10^

The inclusion of a medication in one of the pharmaceutical care programs, for instance, means a captive market in a country where most of the population has no financial resources to afford the costs of medical treatments.^11^ The judicial demand for medications is increasing. Thus, a study addressing the epidemiological profile of these demands is necessary to describe the medications most frequently requested, as well as the most prevalent diseases among these patients. This is additional knowledge that physicians may and should have when prescribing a drug.

## OBJECTIVE

To describe the profile of lawsuits demanding medications filed in the Federal Court.

## METHODS

This is a retrospective descriptive and cross-sectional study assessing the judicial demand of medications filed through the Federal Court of the State of Paraná, between January and December 2014. Lawsuits not originated in the State of Paraná and those whose object was not a medication were excluded. The study was submitted to and approved by the University Research Ethics Committee under number 1.104.832, CAAE: 45493915.7.0000.0093.

Data was obtained by accessing the online database of the Federal Court provided by its administration board, which was previously informed about the study and agreed to it.

The variables analyzed in the study were the plaintiff’s sex and age; city; disease affecting the demandant; brand name of the medication requested; pharmaceutical formulation; family income; total amount of the claim; medical specialty related to plaintiff’s disease; availability in SUS; existing register at ANVISA; use of a public or private defender; date of first court decision; time (in days) elapsed until first court decision; outcome of the first decision (granted or not). We also verified whether the lawsuits contained an interim relief request, which is a type of judicial decision in which the judge should analyze, based on proofs presented by the plaintiff, if the risk of a delay in the judicial proceeding could result in irreparable violation of the plaintiff’s rights. All amounts in reals were converted into American dollars based on the exchange rate on December 30^th^, 2014, when R$ 1,00 was equivalent to US$ 2.66.

### Statistical analysis

Data were tabulated and expressed as medians, means, and standard deviation, or as frequencies and percentages. The statistical analysis was carried out using the Prism 5.0 statistical package (GraphPad Prism, California, USA), and the normality of data was verified using the Kolmogorov-Smirnov tests. The categorical variables were expressed as percentages and compared using the χ^2^ test or Fisher’s exact test, as appropriate. The p values <5% were considered statistically significant.

## RESULTS

A total of 504 lawsuits provided by the database of the Federal Court of the State of Paraná were analyzed. Of these, 347 were eligible for inclusion in the study, and 157 were excluded, according to the established criteria. The most frequent exclusion criterion was lawsuit not related to demand of medication.

Among the 347 plaintiffs, 194 (55.9%) were females with a median age of 56 years (between 1 and 91 years), and 44% of them were aged 60 years or older, 11% were adolescents and 3% were children. Of the lawsuits, 65% were from the city of Curitiba (State of Paraná) and the remaining lawsuits, from cities in the interior of Paraná. The median plaintiff’s monthly family income was US$ 451.12 (R$ 1.200,00), ranging from zero to US$ 5,714.28 (R$ 15.200,00). The plaintiffs declared an income greater than US$ 1,879.69 (R$ 5.000,00) in only five lawsuits.

Table 1 shows the number of lawsuits and amounts involving medications requested through the Federal Court of the State of Paraná in the year of 2014, according to medical specialty and plaintiff’s sex. The medical specialty more frequently involved in the lawsuits was Oncology (23.6%), followed by Pulmonology (15.2%), Rheumatology (14.4%) and Endocrinology (12.1%). Demands from female plaintiffs were more frequently related to medications for rheumatologic diseases (12.4%), whereas those from male plaintiffs were related to medications concerning oncological diseases (13.8%). The total amount of the claim ranged from US$ 112.78 (R$ 300,00) to US$ 331,441.72 (R$ 881.635,00), with a median of US$ 9,398.49 (R$ 25.000,00). The highest total amount per specialty referred to Oncology, distributed into 82 lawsuits, corresponding to 56% of the total amount found in the study. The highest median total amount of the claim was US$ 50,413.15 (R$ 134.099,34), also related to oncologic drugs.

**Table 1:** Lawsuits and amounts regarding medications demanded through the Federal Court, according to medical specialty and plaintiff’s sex

Medical specialties	Lawsuits	Female	Male	Amount (R$)	Amount (US$)*	Median (R$)	Median (US$)*
	(n)	(n)	(n)				
Cardiology	17	7	10	129.113,40	48,538.87	2.500,00	939.84
Dermatology	17	10	7	1.501.916,52	564,630.27	84.000,00	31,578.00
Endocrinology	42	24	18	453.439,14	170,465.84	8.038,98	3,022.17
Hematology	27	11	16	2.138.480,62	803,940.08	79.202,99	29,775.18
Neurology	10	8	2	199.685,08	75,069.57	19.968,51	7,506.95
Ophthalmology	12	8	4	221.576,84	83,299.56	18.464,74	6,941.17
Oncology	82	34	48	10.996.145,93	4,133,889.44	134.099,34	50,789.47
Pulmonology	53	33	20	1.187.246,30	446,333.19	9.899,88	3,721.80
Psychiatry	9	3	6	41.747,24	15,694.45	2.400,00	902.25
Rheumatology	50	43	7	1.540.000,33	578,947.49	25.818,00	9,706.77
Other	28	13	15	975.759,91	366,827.03	34.848,57	13,100.75

Total	347	194	153	19.385.111,31	7,287,635.83	25.000,00	9,398.49

The exchange rate for the American dollar on December 30^th^, 2014, was R$ 1,00 = US$ 2.66.

**Figure 1:**
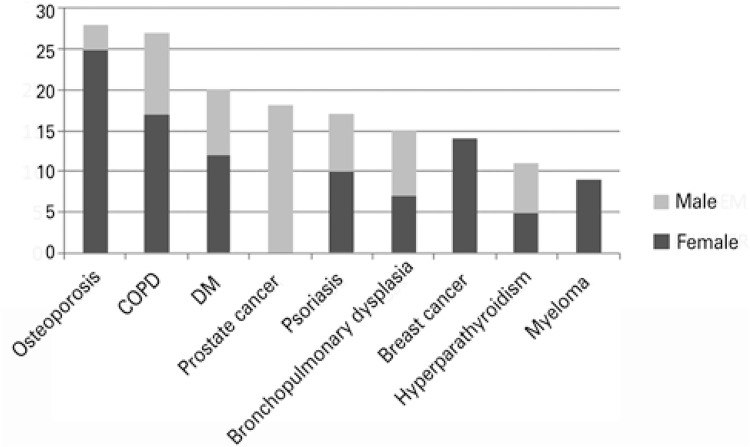
Prevalence of diseases by sex, according to the demand of medications through the Federal Court

COPD: chronic obstructive pulmonary disease; DM: *diabetes mellitus.*

Figure 1 shows the prevalence of diseases by sex. The disease most frequently originating demands was osteoporosis, with 8%, followed by chronic obstructive pulmonary disease (COPD), with 7% of cases, and *diabetes mellitus*, with 5.6%. Among males, prostate cancer was related to the highest number of lawsuits, with 17% of cases, followed by COPD, with 6.5%, and *diabetes mellitus* and bronchopulmonary dysplasia, with 2.4% cases each. Among females, the most frequent disease was osteoporosis, with 12.9% of cases, followed by COPD, with 8.9%, then breast cancer, with 7.3%. A wide range of diseases causing patients to demand medications via lawsuits was observed.

**Figure 2:**
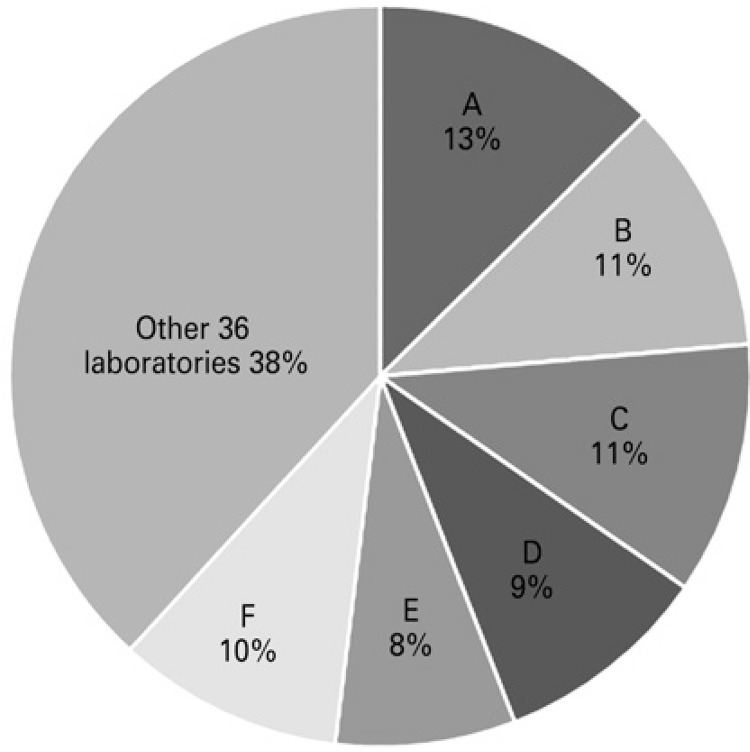
Distribution of medications requested by brand name, per pharmaceutical industry. The names of the industries were replaced by letters, for ethical reasons

Despite the total number of 347 lawsuits, there were 384 medications requested, because in 30 lawsuits more than one type of medication was demanded. Of these 384 medications, 66% were requested by their brand names. Based on the brand name of the medication, we observed that, of 42 pharmaceutical companies producing the medications requested, 6 of them alone accounted for 61.8% of lawsuits (Figure 2).

Table 2 shows a wide range of medications demanded, because in 51.8% of lawsuits the medications could not be grouped by their active ingredient. These cases were categorized as “others” in the table. The medications most frequently requested were tiotropium bromide and palivizumab, both with 6.2% of lawsuits each. These were followed by teriparatide and rituximab, with 3.9% each, and ranibizumab, denosumab, and abiraterone acetate, with 3.6% of lawsuits each. Of the 384 medications requested, only 14.5% (56/384) were registered in the National Medication Registry (*Registro Nacional de Medicamentos* – RENAME). Additionally, in 2.4% (8/384) of cases, the medications were not registered at ANVISA.

**Table 2:** Active ingredients of the medications most frequently required through lawsuits in the Federal Court

Medications	n (%)
Tiotropium bromide	24 (6.3)
Palivizumab	24 (6.3)
Teriparatide	15 (3.9)
Rituximab	15 (3.9)
Ranibizumab	14 (3.6)
Denosumab	14 (3.6)
Abiraterone acetate	14 (3.6)
Insulins	13 (3.4)
Mycophenolate mofetil	11 (2.9)
Cinacalcet	11 (2.9)
Cetuximab	10 (2.6)
Transtuzumab	10 (2.6)
Ustekinumab	10 (2.6)
Others	199 (51.8)

As regards the form of legal representation, 89.6% (311/347) of lawsuits were represented by the Public Defender’s Office. All lawsuits had an interim relief request. In the present study, time for the first court decision in relation to granting the demand ranged from zero to 403 days, with a median of 35 days. The judge in charge of the lawsuit requested expert examination in 46.9% of cases before the first court decision.

In all, 70.0% (243/347) of the judicial demands were granted and 23.9% (83/347) were dismissed. By the end of the present investigation, 6.1% (21/347) of lawsuits had not yet been concluded. Additionally, 0.9% of deaths were observed prior to the decision on the relief, 1.5% of lawsuits were abandoned, and 0.3% of cases were dismissed.

## DISCUSSION

Judicial demands may be either public or private, but most come from public services. These demands may occur both at municipal, state or federal levels. In 1999, only one lawsuit related to medication demand was found at the federal level in the State of Paraná. In 2014, in turn, the year when the present study was conducted, 347 medication demands filed through the Federal Court of the State of Paraná were found. In general, a significant increase in the number of lawsuits is observed at all judicial levels and in several Brazilian States. This reflects a *deficit* in the health system, which is unable to satisfactorily ensure the protection of the fundamental individual right established in Article 196, of the 1988 Brazilian Constitution, that provides “health is a right of all citizen and duty of the State”.^12^

Currently, patients are better informed about their rights regarding health care. Additionally, the courts have become a viable option to solve problems concerning the access to medications and other healthcare-related issues. Thus, the construction of a profile of judicial demands on medications becomes necessary to establish measures to reduce the costs resulting from these lawsuits and to attenuate the stress on patients and their families. Additionally, doctors’ awareness of the diseases more frequently related to the demands, the medications most frequently requested, the costs, and the length of decision in lawsuits filed in the country are believed to be very important for physicians to prescribe and give advice in a more adequate and assertive manner.

When the data of the present study are compared to those of the study conducted by Machado et al.,^10^ we can conclude that the epidemiological profile of plaintiffs had some similarities and some differences. Most of the plaintiffs were females (60.2%). However, we found that 44.3% of plaintiffs were 65 years old or older, whereas in the study by Machado et al.,^10^ 35.4% were elderly individuals.

The diagnosis most frequently found by Machado et al.^10^ and Pereira et al.^4^ was rheumatoid arthritis. Diniz et al.,^13^ in turn, reported that the most prevalent diseases were related to the circulatory and respiratory systems. In our study, osteoporosis was the most prevalent disease related to demands through the Federal Court, although a wide range of conditions originating the demands had been observed.

Chieffi et al.^11^ described that approximately 75% of all lawsuits were conducted by private defenders, whereas only 25% by the Public Defender’s Office. In the present study, we found a different scenario: almost 90% of cases were filed by the Public Defender’s Office, whereas only 10% by private defense. It is noteworthy that the states are in charge of the creation and organization of the Public Defender’s Offices, thus, in some states they have been established very recently. In the State of São Paulo, the Public Defender’s Office was established in 2006; in the State of Paraná, in turn, it exists since 1991 and, as we observed in this study, it has been very busy working on medication demands.

Other aspects to be pointed out are the criteria adopted to recognize the plaintiffs’ poor economic situation required for the use of the Public Defender’s Office, especially as regards the definition of criteria to achieve equal access to SUS and the health system.

Also noteworthy was the presence of the medication in RENAME or not. More than 75% of all medications requested in both studies were not included in this list.^8^

Tiotropium bromide and palivizumab were the medications most often requested in this research. However, some studies report adalimumab as the medication most frequently requested.^11,13^ In our study, this drug accounted for slightly over 1% of total demand. In addition, teriparatide accounted for 3.9% of demands in this study, and was the second medication most frequently requested, along with rituximab. Macedo et al.^12^ found teriparatide as the medication most frequently requested, with 9.9% of all lawsuits. It is important to stress that, in May 2013, the Ministry of Health added adalimumab to the list of pharmaceutical care of SUS for rheumatoid arthritis, psoriatic arthritis, Crohn’s disease and ankylosing spondylitis.

The wide variety of active ingredients of the medications judicially demanded was worth of attention, since in more than half of the lawsuits the medications could not be grouped by active ingredient. Also, among the medications most frequently demanded in the present study, more than half were monoclonal antibodies, and this implicates higher costs.

As regards the costs, the Oncology field showed much higher median values than the other specialties, probably because of the high costs of the medications used for cancer treatment. According to the Ministry of Health,^1^ the costs with lawsuits for the acquisition of medications, equipment, supplies, performance of surgeries and judicial deposits have increased by 500% since 2010. In 2014, the total expenditure with judicialization reached US$ 315,187,969.00 (R$ 838,4 million) in Brazil, including the municipal, state and federal levels. This corresponds to approximately 6% of the amount spent with all medications acquired by the government and distributed by SUS. Our study corroborates these data: only in the Oncology field, we found that the total cost generated with medications was higher than US$ 3,759,398.00 (R$ 10 million), and it accounted for slightly more than 50% of the total amount of claims for the acquisition of medications through the Federal Court of Paraná.

Following the world trend, and particularly because of the increase in life expectancy, a change in the profile of diseases affecting the Brazilian population has been observed and, consequently, an increase in the proportion of malignancies as a cause of morbidity and mortality. The financial burden for the treatment and care of patients with neoplams is high at all stages, from diagnosis to treatment, with increasing use of new technologies, such as monoclonal antibodies. This is undoubtedly a major challenge, especially for universal access health systems, as is the case in Brazil.

Among the medications demanded in lawsuits of this study, most were requested by brand name or trade name in lower amounts than those described by Marques et al.,^14^ which was 77.4%. These figures may partly result from the protection to the current patent, or because they suggest that these lawsuits could include interests of pharmaceutical companies, which trade therapeutic innovations financially inaccessible to the plaintiffs.^11,14^ The fact that most lawsuits determine the supply of a single medication suggests that this path has been used to ensure the access not to pharmaceutical care in general, but to therapeutic novelties, whose costs are usually so high that only the State can afford to buy them.^15,16^ A possible solution for this issue would be a regulation determining that only medications identified by the Common Brazilian Denomination (*Denominação Comum Brasileira*) or, failing that, by the Common International Denomination (*Denominação Comum Internacional*) could be prescribed within the scope of SUS.

As regards the request of interim relief, our findings were similar to those of Ventura et al.,^6^ because all our lawsuits included interim relief request and 70% of the demands were granted. Ventura et al.^6^ found that the judge did not request any other type of document to grant relief in 96.9% of demands. In our study, expert examination was required in 46% of cases, and most were granted.

Most of the time, the judges, laymen in terms of medical issues, rely on documents provided by the plaintiffs, which are mainly focused on prescriptions and other medical documents to request interim relief, indicating possible urgency and need for the medication. Thus, most of the times, the judge grants urgent relief and the citizen (patient and plaintiff) can start taking the medication, and only later a decision will be made on whether this medication was indeed necessary.

Many factors may negatively influence the quality of medical prescriptions. With heavy investments in advertising, the pharmaceutical industries may exert great influence on the prescription of a medication, and this can be confirmed by the large number of prescriptions using the brand name. The lack of efficient control tools on the veracity of information makes this situation even worse.^17^

## CONCLUSION

Most of the plaintiffs demanding medications through the Federal Court of the State of Paraná in the year of 2014 were females, with a median age of 56 years and residing in the city of Curitiba. The medications requested were mostly from the Oncology field and the condition most frequently related to the lawsuits was osteoporosis, although a wide range of medications had been requested. Approximately two thirds of medicines were requested by their brand names. Palivizumab and tiotropium bromide are among those most frequently requested. The minority of medications was included in the National Medication Registry, and the large majority was registered at ANVISA. Additionally, most of the lawsuits were filed by the Public Defender’s Office. All demands required interim relief, and most were granted within a mean time of 35 days.

There is a current trend for increased judicial request of medications, and further studies on judicial demands are necessary. These studies should assess three areas: how the lawsuit is conducted and who will judge it; the way medical prescriptions are made; and which medications should be part of the list of the National Medication Registry. Judicial demands for increasingly more expensive medicines is a reality, and a scenario of need for greater investments emerges in contrast to limited resources and a mandatory search for more effective and efficient strategies.
